# Supplementary material for: Enhanced disulphide bond stability contributes to the once-weekly profile of insulin icodec
Source: Nat Commun. 2024 Jul 20;15:6124. doi: 10.1038/s41467-024-50477-9 (PMC11271312; doi:10.1038/s41467-024-50477-9)
Supplement: Supplementary file 1 — Supplementary information [file 41467_2024_50477_MOESM1_ESM.docx]

**Supplementary Figure 1**. Structures of compounds

Insulin icodec


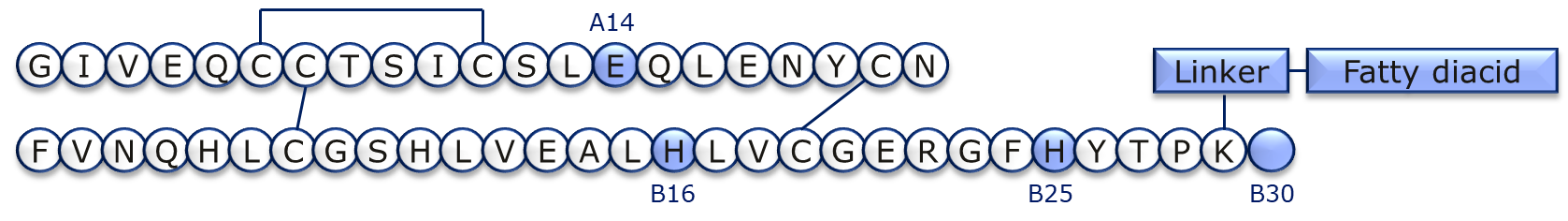


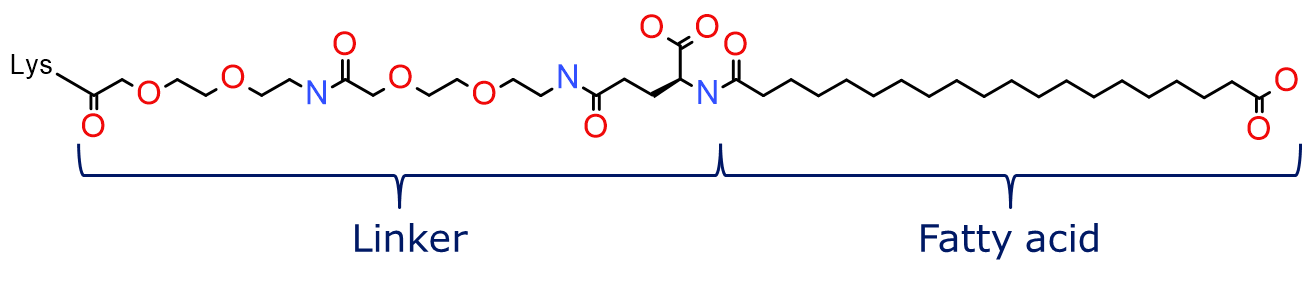


**Supplementary Table 1. Structures of insulin analogs studied in article.**

| Label | Substitutions relative to HI* | Fatty acid |
| --- | --- | --- |
| C18 | desB30 | C18 diacid |
| B16H C18 | B16H, desB30 | C18 diacid |
| B25H C18 | B25H, desB30 | C18 diacid |
| A14E C18 | A14E, desB30 | C18 diacid |
| A14E, B25H C18 | A14E, B25H, desB30 | C18 diacid |
| A14E, B25H C20 | A14E, B25H, desB30 | C20 diacid |
| Insulin Icodec | A14E, B16H, B25H, desB30 | C20 diacid |

*human insulin contains Tyr in positions A14 and B16, Phe in position B25 and Thr in position B30.

All compounds contain the same linker as insulin icodec.

**Supplementary Figure 2.** **HPLC chromatograms and MS spectra for insulin degradation products identified under different redox conditions.**

**a.** High-performance liquid chromatography chromatograms showing human insulin after 4-h incubations at 37 °C under different redox conditions as described in the Methods section. Top panel represents incubation of human insulin without added glutathione, Middle panel shows human insulin incubated with 0.625 mM GSH and 1 mM GSSG approximately representing a state where 50% of HI is degraded and Bottom panel shows human insulin incubated with 6.3 mM GSH and 1 mM GSSG. **b.** Structures of selected species from panel a. **c.** Mass spectra of different A-chain species. **d.** Mass spectra of different B-chain species.

**a**

**b**


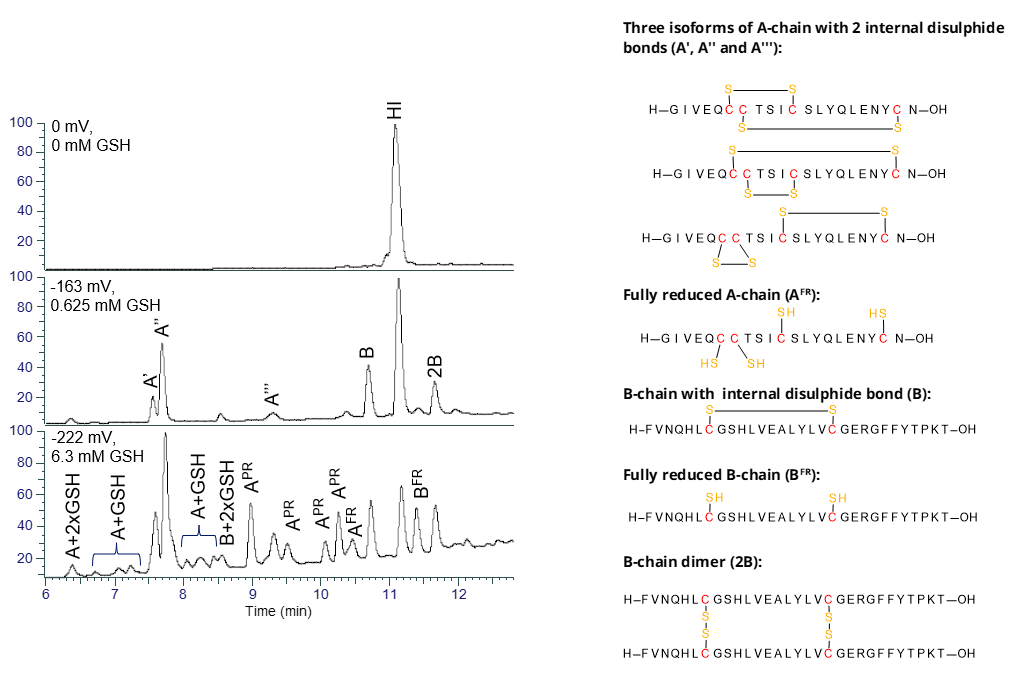


**c**


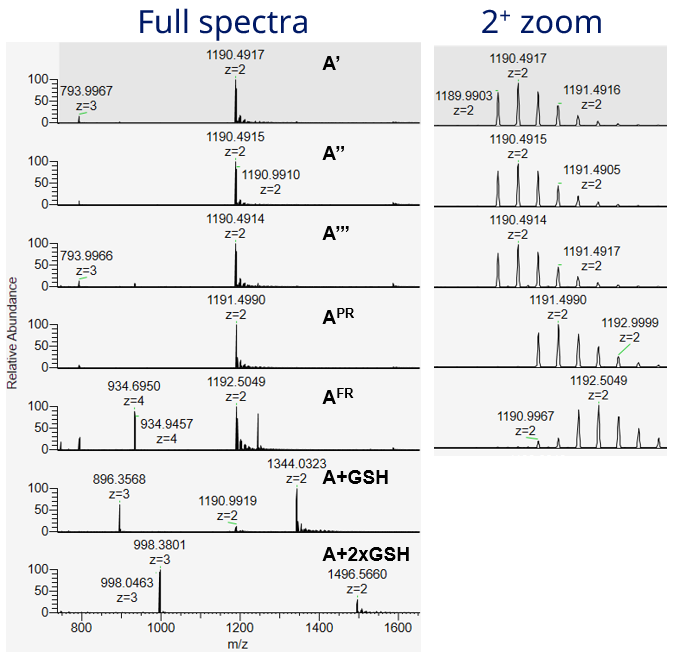


**d**


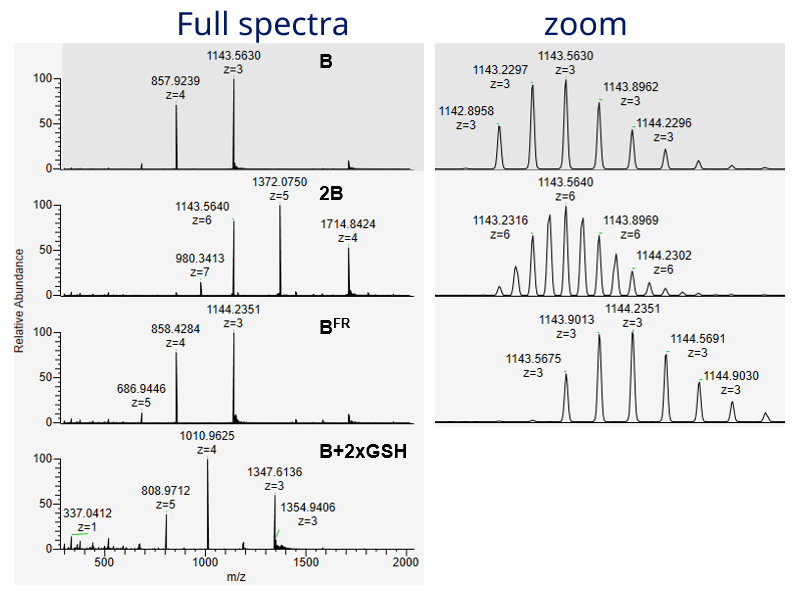


**Supplementary Table 2. Summary of observed masses in Supplementary Figure 2.**

| Name | Description | RT (min) | m/z | z | M_monoisotopic_  (Da) | Mass difference from a reference* |
| --- | --- | --- | --- | --- | --- | --- |
| A+2GSH | A-chain attached to two glutathione molecules via disulphide bonds and containing an internal disulphide bond | 6.5 | 997.7124 | 3 | 2990.115 | 612.1467 |
| A+GSH | A-chain isoforms attached to one glutathione molecule via disulphide bond and containing an internal disulphide bond | 7-7.3 | 1343.5312 | 2 | 2685.048 | 307.0791 |
| A' | A-chain isoform containing 2 disulphide bonds | 7.6 | 1189.9903 | 2 | 2377.966 | -0.0027 |
| A'' | A-chain isoform containing 2 disulphide bonds | 7.7 | 1189.9899 | 2 | 2377.965 | -0.0035 |
| A+GSH | A-chain isoforms attached to one glutathione molecule via disulphide bond and containing an internal disulphide bond | 8.15-8.65 | 1343.5310 | 2 | 2685.047 | 307.0787 |
| B+2GSH | B-chain attached to two glutathione molecules via disulphide bonds | 8.5 | 1010.4621 | 4 | 4037.819 | 612.1504 |
| A^PR'^ | Partially reduced A-chain isoform containing a disulphide bond | 9 | 1190.9986 | 2 | 2379.983 | 2.0139 |
| A''' | A-chain isoform containing 2 disulphide bonds | 9.4 | 1189.9894 | 2 | 2377.964 | -0.0045 |
| A^PR'^' | Partially reduced A-chain isoform containing a disulphide bond | 9.5 | 1190.9978 | 2 | 2379.981 | 2.0123 |
| A^PR'^'' | Partially reduced A-chain isoform containing a disulphide bond | 10.1 | 1190.9974 | 2 | 2379.98 | 2.0115 |
| A^PR'^''' | Partially reduced A-chain isoform containing a disulphide bond | 10.3 | 1190.9985 | 2 | 2379.982 | 2.0137 |
| A^FR^ | Fully reduced A-chain | 10.5 | 1192.0037 | 2 | 2381.993 | 4.0241 |
| B | A-chain containing disulphide bond | 10.7 | 1142.8958 | 3 | 3425.666 | -0.0033 |
| HI | human insulin | 11.1 | 1161.7350 | 5 | 5803.639 | 0.0010 |
| 2B | B-chain dimer via two disulphide bonds | 11.7 | 1371.2746 | 5 | 6851.337 | -0.0012 |
| B^FR^ | Fully reduced B-chain | 11.7 | 1143.5675 | 3 | 3427.681 | 2.0118 |

*Monoisotopic mass of A-chain including internal disulfide bonds (2377.9687 Da) is used as a reference for all A-chain forms. Monoisotopic mass of B-chain including internal disulfide bond (3425.6689 Da) is used as a reference for all B-chain forms. Monoisotopic mass of human insulin (5803.6376 Da) was used for HI.

**Supplementary Figure 3.** **Far-UV circular dichroism spectra.** Far-UV CD spectra of 5 µM insulin analogs recorded in 10 mM Tris/HClO4, pH 8.0, n=1. Source data are provided as a Source Data file.

The shape and intensity of the far-UV circular dichroism spectrum (FUV-CD) of insulin provide a fingerprint of the protein back-bone folding. Typically, insulin analogues display overall spectral features; two minima at 208 nm and at 222 nm, and a maximum at 195 nm. The spectra for the insulins used for the study presented in FIG are quantitatively very similar but with a slight variation in the intensities of the 208nm and 222 nm bands. The variation in intensities are within expected error cause in determination of the concentration of the test solution. The noise of the spectra in the 195-200 nm range is due to interfering buffer components

**Supplementary Figure 4.** **Human insulin *in vitro* plasma stability incubations in plasma from rats.** **a** disappearance of intact human insulin over time, n=2 and **b** identified degradation products of human insulin in rat plasma. All identified metabolites are proteolytic degradation product, with no detection of the free B-chain. Nomenclature example: A1-13_B1-9 refers to the product consisting of A-chain amino acids 1-13 being connected to the B-chain amino acids 1-9 through the interchain disulfide bonds connecting these peptides, n=2. Source data are provided as a Source Data file.

**Supplementary Figure 5.** B**-chain formation during *in vitro* plasma incubations of various insulin analogs in plasma from humans (a), dogs (b) and minipigs (c)**, n=2. All insulin B-chains contain an internal disulphide bond connecting Cys 7 with Cys 19, assessed by the exact monoisotopic masses. Co-incubations were used in these experiments, where the C18 and A14E, C18 analogs containing the same B-chain were grouped and the B25H, C18 and A14E, B25H, C18 analogs also containing the same B-chain were grouped, and thus the plotted values are the summed observed B-chain levels of these pairs of analogs. Source data are provided as a Source Data file.

**Supplementary Figure 6. Stereo view of a representative part of the electron density.**  2Fo-Fc density is contoured at 1 sigma.  Amino acids are in stick representation. Shown is how Arg 22 of icodec's B-chain covers the intra-chain disulfide bond formed by Cys A20 and Cys B19.


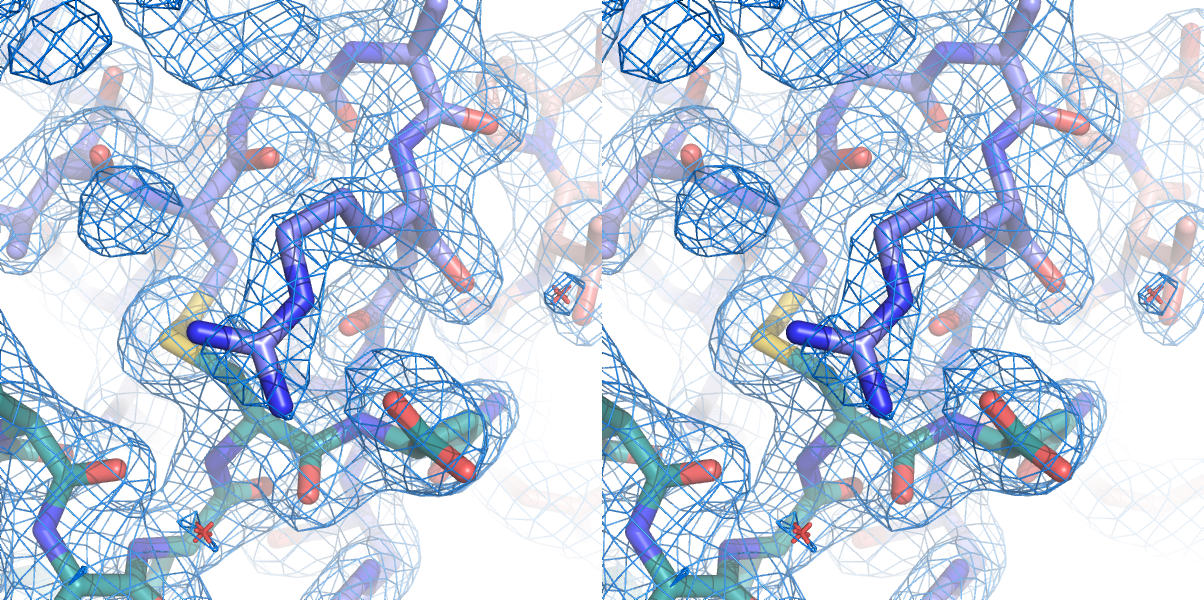


**Supplementary Table 3. Metabolite identification data and methods from LC-MS analysis of human serum and matching to metabolite structures in manuscript Figure 6b**

| **Selected time points** | **Analytes** | **MS-peak retention time (min)** | | **^A^****Mass of protonated ion** | | **^B^Type of ion** | **^C^Mass in neutral state (Da)** | | **Chemical formula from proposed structure** | | **^C^Mass of proposed structure (Da)** | **^D^Mass Accuracy (****ppm)** |
| --- | --- | --- | --- | --- | --- | --- | --- | --- | --- | --- | --- | --- |
| 168 h after 5th dose | **Insulin icodec** | 21.78 | | 6377.0244 | | [M+H^+^]^+^ | 6376.0171 | | C_280_H_435_N_71_O_87_S_6_ | | 6376.0122 | -0.8 |
|  | **B-Chain** | 22.81 | | 4033.0735 | | [M+H^+^]^+^ | 4032.0662 | | C_185_H_286_N_46_O_51_S_2_ | | 4032.0642 | -0.5 |
|  | **B24-29** | 27.20 | | 1535.8705 | | [M+H^+^]^+^ | 1534.8632 | | C_76_H_118_N_12_O_21_ | | 1534.8534 | -6.4 |
| 348 h after 5th dose | **Insulin icodec** | 21.78 | | 6377.0225 | | [M+H^+^]^+^ | 6376.0152 | | C_280_H_435_N_71_O_87_S_6_ | | 6376.0122 | -0.5 |
|  | **B-Chain** | 22.83 | | 4033.0757 | | [M+H^+^]^+^ | 4032.0684 | | C_185_H_286_N_46_O_51_S_2_ | | 4032.0642 | -1.0 |
|  | **B24-29** | 27.16 | | 1535.8634 | | [M+H^+^]^+^ | 1534.8561 | | C_76_H_118_N_12_O_21_ | | 1534.8534 | -1.8 |
|  | **B29** | 28.43 | | 890.5681 | | [M+H^+^]^+^ | 889.5608 | | C_43_H_79_N_5_O_14_ | | 889.5624 | 1.8 |
| ^A^Monoisotopic mass of [M+H]^+^ ions obtained from following MaxEnt3 deconvolution of multiply charged ions  ^B^The monoisotopic masses of H^+^ used in the calculation of M was 1.0073  ^C^Monoisotopic mass in neutral state (M)  ^D^Mass accuracy = [(proposed mass - M)/M] x 1x10^6^ | | | | | | | | | | | | |
| **Column, mobile phase and settings** | | | | | | | | | | | | |
| Column | | | Peptide BEH C_18_ column, 300Å, 2.1x150 mm, 1.7 µm (Waters), | | | | | | | | | |
| Mobile phase | | | A: formic acid:water 1:99 (v/v) and B: formic acid:acetonitrile:water 1:90:10 (v/v/v) | | | | | | | | | |
| Inlet method – Autosampler | | | Column temperature: 50 °C | | | | | | | | | |
| The serum samples were injected with repeated injections of 10 μl prior to injection of another 10 μl (total injection volumes of 40 μl).followed by start of the mobile phase gradient below | | | | | | | | | | | | |
| Inlet method – Inlet | | | Time (min) | | Flow rate (ml/min) | | | %A | | %B | | |
|  | | | 0 | | 0.4 | | | 95 | | 5 | | |
|  | | | 10 | | 0.4 | | | 70 | | 30 | | |
|  | | | 25 | | 0.4 | | | 55 | | 45 | | |
|  | | | 35 | | 0.4 | | | 30 | | 70 | | |
|  | | | 40 | | 0.4 | | | 0 | | 100 | | |
|  | | | 40.1 | | 0.4 | | | 95 | | 5 | | |
|  | | | 45 | | 0.4 | | | 95 | | 5 | | |
| **MS settings for metabolite identification analysis** | | | | | | | | | | | | |
| Capillary voltage: 2.5 kV, sampling cone: 40, source offset: 50, source temperature: 150°C, desolvation temperature: 550°C, desolvation gas flow: 1000 l/h, nebuliser gas flow: 6.0, analyser mode (operation mode): Resolution, start and end times: 0 and 35 min, polarity (ion mode): Positive, scan time: 1 sec, data format: Continuum, low and high mass (mass interval): 500 and 1500 Da, trap and transfer collision energies 4 V. | | | | | | | | | | | | |
| Lock mass: Leucine Enkephalin (200 pg/μl in formic acid:acetonitrile:water 1:500:500 (v/v/v)) | | | | | | | | | | | | |

**Supplementary Table 4. Quantitative data and methods from LC-MS/MS analysis of human serum with concentrations of insulin icodec and its metabolites**

|  |  | |  | **Serum concentrations (pmol/L)** | | | | | | | | |
| --- | --- | --- | --- | --- | --- | --- | --- | --- | --- | --- | --- | --- |
| **Dosing at  Trial Day** | **Number of  weekly doses** | | **Analytes** | **0 h** | | **10 h** | **20 h** | | **168 h** | **348 h** | | **516 h** |
| 1 | 1 | | **insulin icodec** | <LLOQ | | 227000 | 225000 | | 107000 | - | | - |
|  |  |  | **B-chain** | <LLOQ | | <LLOQ | 5460 | | 15900 | - | | - |
|  |  |  | **B24-29** | <LLOQ | | <LLOQ | <LLOQ | | <LLOQ | - | | - |
|  |  |  | **B29** | <LLOQ | | 2980 | 4170 | | 4350 | - | | - |
| 29 | 5 | | **insulin icodec** | 215000 | | NA | 493000 | | 240000 | 95500 | | 47100 |
|  |  |  | **B-chain** | 39300 | | NA | 46600 | | 47900 | 27500 | | 14100 |
|  |  |  | **B24-29** | 2880 | | NA | 3110 | | 2990 | 2710 | | 2690 |
|  |  |  | **B29** | 8790 | | NA | 10900 | | 11200 | 8490 | | 7060 |
| NA = not analysed, - = not sampled  LLOQ for insulin icodec = 10000 pmol/L  LLOQ for B-chain = 2000 pmol/L  LLOQ for B24-29 = 1000 pmol/L  LLOQ for B29 = 1000 pmol/L | | | | | | | | | | | | |
| **Quantitative analysis** | | | | | | | | | | | | |
| Calibration standards were prepared in human blank serum for the following reference substances: insulin icodec, insulin icodec B-chain, B24-29 metabolite and B29 metabolite. After analysis, the area under the curves of peaks from insulin icodec and metabolites were obtained by peak integration with the QuanLynx application in MassLynx 4.1. | | | | | | | | | | | | |
| **Column, mobile phase and settings** | | | | | | | | | | | | |
| Column | | Peptide BEH C_18_ column, 300Å, 2.1x150 mm, 1.7 µm (Waters), | | | | | | | | | | |
| Mobile phase | | A: formic acid:water 1:99 (v/v) and B: formic acid:acetonitrile:water 1:90:10 (v/v/v) | | | | | | | | | | |
| Inlet method – Autosampler | | Column temperature: 50 °C | | | | | | | | | | |
| Inlet method – Inlet | | Time (min) | | | Flow rate (ml/min) | | | %A | | | %B | |
|  | | 0 | | | 0.4 | | | 95 | | | 5 | |
|  | | 1 | | | 0.4 | | | 70 | | | 30 | |
|  | | 5 | | | 0.4 | | | 55 | | | 45 | |
|  | | 8 | | | 0.4 | | | 30 | | | 70 | |
|  | | 10 | | | 0.4 | | | 0 | | | 100 | |
|  | | 10.1 | | | 0.4 | | | 95 | | | 5 | |
|  | | 15 | | | 0.4 | | | 95 | | | 5 | |
| **MS/MS settings** | | | | | | | | | | | | |
| The same MS settings as those from metabolite analysis were used, with the exception from the MRM transition settings, with selected precursor ions (Q1), product ions (TOF) and retention times (tR):   - Insulin icodec: *m/z* 1064 ® 600.3 (Q1®TOF), tR = 6.1 ± 0.1 min - NNC0148-3204 (B-chain): *m/z* 807 ® 600.3 (Q1®TOF), tR = 6.4 ± 0.1 min - NNC0090-4221 (B24-29): *m/z* 768.4 ® 987.6 (Q1®TOF), tR = 7.7 ± 0.1 min - NNC0099-0589 (B29): *m/z* 890.5 ® 642.4 (Q1®TOF), tR = 8.1 ± 0.1 min | | | | | | | | | | | | |

**Supplementary Table 5.** **Pharmacokinetic parameters from human serum concentrations of insulin icodec and its metabolites**

| **Dosing at  Trial Day** | **Number of weekly doses** | **Analytes** | **t_max_ (h)** | **C_max_ (pmol/L)** | **t_last_ (h)** | **AUC_last_  (h x pmol/L)** | **AUC_0-168h_  (h x pmol/L)** | **% of total  AUC_last_** | **% of total**  **AUC_0-168h_** |
| --- | --- | --- | --- | --- | --- | --- | --- | --- | --- |
| 1 | 1 | **insulin icodec** | 10 | 227000 | 168 | 26900000 | 26900000 | 92.1 | 92.1 |
|  |  | **B-chain** | 168 | 15900 | 168 | 1640000 | 1640000 | 5.60 | 5.60 |
|  |  | **B24-29** | NC | NC | NC | NC | NC | NC | NC |
|  |  | **B29** | 168 | 4350 | 168 | 681000 | 681000 | 2.33 | 2.33 |
| 29 | 5 | **insulin icodec** | 20 | 493000 | 516 | 98800000 | 59100000 | 80.3 | 85.3 |
|  |  | **B-chain** | 168 | 47900 | 516 | 17800000 | 7850000 | 14.5 | 11.3 |
|  |  | **B24-29** | 20 | 3110 | 516 | 1480000 | 511000 | 1.20 | 0.740 |
|  |  | **B29** | 168 | 11200 | 516 | 4900000 | 1830000 | 3.98 | 2.64 |
| NC = not calculated due to lacking concentration data  Concentration data rounded to three significant numbers | | | | | | | | | |
| **Pharmacokinetic calculations** | | | | | | | | | |
| The serum concentration versus time data was evaluated by non-compartmental analysis (NCA) in PhoenixTM WinNonlin® version 6.4, build 6.4.0.768 (Pharsight®, St. Louis, Missouri, USA). Nominal sampling times were used, and the concentration values were used with full precision. The AUCs were calculated using the “Linear Up Log Down” method, *i.e*. the linear trapezoidal rule when the concentration increased, and the logarithmic trapezoidal rule when the concentration decreased. | | | | | | | | | |
