# Supplementary material for: Enhanced disulphide bond stability contributes to the once-weekly profile of insulin icodec
Source: Nat Commun. 2024 Jul 20;15:6124. doi: 10.1038/s41467-024-50477-9 (PMC11271312; doi:10.1038/s41467-024-50477-9)
Supplement: Supplementary file 3 — Reporting Summary [file 41467_2024_50477_MOESM3_ESM.pdf]

nature portfolio

Corresponding author(s): Frantisek Hubalek

Last updated by author(s): Jun 16, 2024

Reporting Summary

Nature Portfolio wishes to improve the reproducibility of the work that we publish. This form provides structure for consistency and transparency in reporting. For further information on Nature Portfolio policies, see our Editorial Policies and the Editorial Policy Checklist.

Statistics

For all statistical analyses, confirm that the following items are present in the figure legend, table legend, main text, or Methods section.

n/a	Confirmed	
☐	☒	The exact sample size (*n*) for each experimental group/condition, given as a discrete number and unit of measurement
☒	☐	A statement on whether measurements were taken from distinct samples or whether the same sample was measured repeatedly
☒	☐	The statistical test(s) used AND whether they are one- or two-sided<br>*Only common tests should be described solely by name; describe more complex techniques in the Methods section.*
☒	☐	A description of all covariates tested
☒	☐	A description of any assumptions or corrections, such as tests of normality and adjustment for multiple comparisons
☐	☒	A full description of the statistical parameters including central tendency (e.g. means) or other basic estimates (e.g. regression coefficient) AND variation (e.g. standard deviation) or associated estimates of uncertainty (e.g. confidence intervals)
☒	☐	For null hypothesis testing, the test statistic (e.g. *F*, *t*, *r*) with confidence intervals, effect sizes, degrees of freedom and *P* value noted<br>*Give P values as exact values whenever suitable.*
☒	☐	For Bayesian analysis, information on the choice of priors and Markov chain Monte Carlo settings
☒	☐	For hierarchical and complex designs, identification of the appropriate level for tests and full reporting of outcomes
☒	☐	Estimates of effect sizes (e.g. Cohen's *d*, Pearson's *r*), indicating how they were calculated

Our web collection on statistics for biologists contains articles on many of the points above.

Software and code

Policy information about availability of computer code

Data collection	MassLynx v4.1 (Waters Inc., Milford, MA, USA); Xcalibur v4.4.16.14 (Waltham, MA, USA)
Data analysis	Graph Pad Prism, version 9.0.1 (GraphPad Software, Boston, MA, USA); PhoenixTM WinNonlin® version 6.4, build 6.4.0.768 (Pharsight®, St. Louis, Missouri, USA); Coot (Version 0.8.9); Phenix suite of programs version 1.20.1 (https://phenix-online.org/)

For manuscripts utilizing custom algorithms or software that are central to the research but not yet described in published literature, software must be made available to editors and reviewers. We strongly encourage code deposition in a community repository (e.g. GitHub). See the Nature Portfolio guidelines for submitting code & software for further information.

Data

Policy information about availability of data

All manuscripts must include a data availability statement. This statement should provide the following information, where applicable:

- Accession codes, unique identifiers, or web links for publicly available datasets
- A description of any restrictions on data availability
- For clinical datasets or third party data, please ensure that the statement adheres to our policy

All data associated with this work are present in the paper. Source data are provided with this paper.
X-ray structures with the following access codes were used in this study:
6S4I http://dx.doi.org/10.2210/pdb6s4i/pdb

6S34 http://dx.doi.org/10.2210/pdb6s34/pdb
Crystal structure coordinates and structure factors for insulin icodec structure were deposited in the Protein Data Bank (PDB) under the following accession codes: 8RRP. https://pdbj.org/search/status?pdbid=8rrp
The authors declare that the data supporting the findings of this study are available within the manuscript.

Research involving human participants, their data, or biological material

Policy information about studies with human participants or human data. See also policy information about sex, gender (identity/presentation), and sexual orientation and race, ethnicity and racism.

Reporting on sex and gender	Reporting of data from pools of residual PK serum selected from 12 male human subjects. The exploratory metabolite analysis was described in the clinical trial report (Novo Nordisk Trial ID: NN1436-4314, ClinicalTrials.gov ID: NCT02964104), and documented independently and reported separately.
Reporting on race, ethnicity, or other socially relevant groupings	Human subjects with type 2 diabetes mellitus
Population characteristics	Key inclusion criteria (Novo Nordisk Trial ID: NN1436-4314, ClinicalTrials.gov ID: NCT02964104)<br>- Male or female, age between 18 and 64 years (both inclusive) at the time of signing informed consent.<br>- Subject who is considered to be generally healthy (with the exception of conditions associated with diabetes mellitus), based on the medical history, physical examination, and the results of vital signs, ECG and laboratory safety tests, as judged by the investigator.<br>- Body mass index between 20.0 and 34.9 kg/m2 (both inclusive).<br>- Type 2 diabetes mellitus (as diagnosed clinically) for ≥12 months (365 days).<br>- Treated with or without any metformin formulations (dose documented in the subject medical record). If treated with metformin, the total daily dose of metformin must have been stable within the past 90 days prior to the day of screening.<br>- No change in insulin treatment regimen during the last 90 days prior to screening.<br>- Current total daily insulin treatment between 0.3 and 1.0 (I) U/kg/day (both inclusive).<br>- HbA1C ≤9.0% based on central laboratory analysis
Recruitment	Screening of subjects based on baseline demographic and disease characteristics
Ethics oversight	Trial protocol, amendments, subject information and consent forms were reviewed and approved by an independent ethical committee Ärztekammer Nordrhein, Körperschaft des öffentlichen Rechts, Ethikkommission, Germany

Note that full information on the approval of the study protocol must also be provided in the manuscript.

Field-specific reporting

Please select the one below that is the best fit for your research. If you are not sure, read the appropriate sections before making your selection.

☒ Life sciences ☐ Behavioural & social sciences ☐ Ecological, evolutionary & environmental sciences

For a reference copy of the document with all sections, see nature.com/documents/nr-reporting-summary-flat.pdf

Life sciences study design

All studies must disclose on these points even when the disclosure is negative.

Sample size	Sample size in these preclinical studies was chosen to provide reasonable number of replicates (n=3 minipigs per group; it was not considered justifiable to use larger group of minipigs for preclinical studies).
Data exclusions	No data points were excluded
Replication	No study replication
Randomization	No randomization
Blinding	No blinding

Reporting for specific materials, systems and methods

We require information from authors about some types of materials, experimental systems and methods used in many studies. Here, indicate whether each material, system or method listed is relevant to your study. If you are not sure if a list item applies to your research, read the appropriate section before selecting a response.

Materials & experimental systems

n/a	Involved in the study
☒	☐ Antibodies
☒	☐ Eukaryotic cell lines
☒	☐ Palaeontology and archaeology
☐	☒ Animals and other organisms
☒	☐ Clinical data
☒	☐ Dual use research of concern
☒	☐ Plants

Methods

n/a	Involved in the study
☒	☐ ChIP-seq
☒	☐ Flow cytometry
☒	☐ MRI-based neuroimaging

Animals and other research organisms

Policy information about studies involving animals; ARRIVE guidelines recommended for reporting animal research, and Sex and Gender in Research

Laboratory animals	Healthy conscious Ellegaard Göttingen Minipigs, weighing approximately 22 kg
Wild animals	Wild animals were not used in this study
Reporting on sex	Three female minipigs were used in this study. Similar results would be expected for both male and female minipigs. Female minipigs were selected for the study as they typically exhibit nicer behavior in the stables compare to the male minipigs.
Field-collected samples	No field collected samples were used in this study
Ethics oversight	Animal experiments were performed according to permission granted by the Animal Experiments Inspectorate, Ministry of Environment and Food of Denmark.

Note that full information on the approval of the study protocol must also be provided in the manuscript.

Plants

Seed stocks	Seed stocks were not used in this study
Novel plant genotypes	Novel plant genotypes were not used in this study
Authentication	*Describe any authentication procedures for each seed stock used or novel genotype generated. Describe any experiments used to assess the effect of a mutation and, where applicable, how potential secondary effects (e.g. second site T-DNA insertions, mosiacism, off-target gene editing) were examined.*
